# Observations on Variations in Manual Reading of Cultures

**DOI:** 10.1128/JCM.01380-16

**Published:** 2016-10-24

**Authors:** John Glasson, Rhys Hill, Michael Summerford, Steven Giglio

**Affiliations:** a LBT Innovations Ltd., Adelaide, South Australia, Australia; b Australian Centre for Visual Technologies, University of Adelaide, South Australia, Australia; c Clinpath Laboratories, Kent Town, South Australia, Australia; Brigham and Women's Hospital

## LETTER

The subject of laboratory error was examined during a symposium at the ASM Microbe 2016 conference in Boston, MA. During the session, it became clear that discussion on this subject has not flourished within our field, possibly due to a lack of published experiences. We therefore submit our observations from a recent series of trials in which we evaluated an image analysis device (1) by comparing results from panels of microbiologists. The variations between members of the panels are reported here.

Urine cultures were prepared by inoculating sheep blood and MacConkey agars (Remel, Lenexa, KS, USA) at one laboratory in the United States and two in Australia. Following the incubation, three microbiologists at each site with demonstrated competency in the reading of urine cultures independently recorded the quantity of growth on each plate as 0, 10^3^, 10^4^, or ≥10^5^ CFU/ml. Colony morphologies were also reported using defined criteria. A decision regarding the designation of each case as positive (i.e., a case requiring further work such as isolate identification and antibiotic susceptibility testing) or negative (i.e., a case without growth or with probable contaminants that did not require further work) was recorded after the application of an interpretive rule set (2). A consensus result, defined as agreement between two or more panel member readings, was used as the “gold standard” for comparative purposes.

In total, 10,077 urine samples were tested and provided 30,231 blood agar and 30,231 MacConkey plate readings. Within the panels, there was 94.5% (28,565/30,231) agreement for the level of growth from blood agar, with 1,666 readings that differed from the consensus. Of these, 936 were reported as lower counts and 730 reported as higher. With MacConkey agar, agreement for the level of growth was 98.5% (29,674/30,231), with 557 divergent readings, 332 being reported as lower counts and 225 reported as higher.

A counting error of 6.6% has been reported by others (3), and our findings reinforce their view that growth enumeration is not always accurate and that the manual assessment of colony numbers provides only an estimate of colony growth (3, 4).

For the reporting of the different colony morphologies, we noted 87.5% (14,912/17,034) agreement by the panel members with the blood agar isolates and 97.5% (18,302/18,774) with the MacConkey agar. For the final designation of each case, we observed 96.4% (29,131/30,231) agreement among the panel members, with 1,100 differences being noted. Of these, 46.3% (509/1,100) were determined to be positive when the panel consensus was negative and 53.7% (591/1,100) negative when the panel consensus was positive. There were no significant differences in any of the proportions of variations between the sites.

Our findings demonstrate that reported data can differ, even when experienced microbiologists apply defined rule sets. As these differences have the potential to impact patient care, a better understanding of their incidence and importance may further assist us in reducing laboratory errors. The findings also suggest that a cautious approach is advisable when consensus results from panels of microbiologists are used as the gold standard in the assessment of new technologies.
